# The Prevalence of Pulmonary Embolism in Patients With Interstitial Lung Disease: A Cross-Sectional Retrospective Study

**DOI:** 10.7759/cureus.23063

**Published:** 2022-03-11

**Authors:** Rahmah Alsilmi

**Affiliations:** 1 Pulmonary Medicine, King Abdulaziz University, Jeddah, SAU

**Keywords:** anticoagulant, idiopathic pulmonary fibrosis, venous thromboembolism, pulmonary embolism, interstitial lung disease

## Abstract

Objective: Interstitial lung disease (ILD) can be complicated by comorbidities, particularly pulmonary embolism (PE). We aimed to assess the prevalence of PE in ILD patients.

Methods: Our study is a cross-sectional retrospective study conducted on ILD cases diagnosed between January 1, 2010, and June 30, 2021. Out of the total ILD cases (n = 153), we enrolled for analysis only those who underwent a computed tomography pulmonary angiography (CTPA) (n = 48). We recorded the number of patients who had a PE event on CTPA, gender, age at PE and ILD diagnoses, a chronology of PE with ILD diagnosis, PE characteristics, PE therapy, type of ILD, radiographic progression of ILD, presence of pulmonary hypertension, and mortality.

Results: Seven patients out of 48, had PE (14.6%). The mean age at the time of PE diagnosis was 70 ± 9.73 years. No statistical difference existed between the PE and non-PE groups regarding gender predominance or the age at ILD diagnosis. All of the identified PE events (n = 7) were segmental (100%), one was saddle PE (14.3%) and one was recurrent (14.3%). No PE events were diagnosed prior to ILD diagnosis, three patients (42.9%) had a simultaneous diagnosis of PE and ILD, and four patients (57.1%) were diagnosed with a PE after ILD diagnosis by a mean time of eight months. No difference in ILD radiographic progression, pulmonary hypertension, or mortality between the two groups was found.

Conclusion: PE is not uncommon in ILD and needs to be ruled out, especially in patients with worsening respiratory status.

## Introduction

Interstitial lung diseases comprise varied disease patterns, including idiopathic interstitial pneumonia (such as idiopathic pulmonary fibrosis, IPF), ILDs with underlying etiologies (such as connective tissue disease-associated ILD, CTD-ILD), exposure-related ILDs (such as hypersensitivity pneumonitis, HP), and granulomatous ILDs (such as sarcoidosis) [1]. ILD poses challenges regarding diagnosis and management, and because clinical behavior, response to treatment, and prognosis vary among different ILDs, it is of utmost importance to make a proper diagnosis. In addition, comorbid medical conditions should be carefully screened for and diagnosed, such as pulmonary embolism, pulmonary hypertension (PH), emphysema, lung cancer, coronary artery disease, gastroesophageal reflux disease, and sleep-disordered breathing. These comorbidities, which have been reported in patients with ILD at different prevalence rates, can contribute to morbidity and mortality [2,3]. Pulmonary embolism (PE) can lead to significant deterioration of a patient’s condition, including the development of respiratory failure and PH, which affect prognosis and lead to mortality. Thus, PE should be included in the differential diagnosis when a patient with ILD presents with acute or gradual worsening of respiratory status, newly developed PH, or hypoxemia that is out of keeping with the degree of parenchymal lung disease for which it is important to investigate for PE, as it is considered a potentially treatable condition. Therefore, when ILD patients present with a change in their respiratory status, it is necessary to use CTPA to rule out PE before labeling the condition as an acute exacerbation of ILD (AEILD), which has a worse prognosis than PE. The PE event rate in ILD has been estimated in previous studies to range between 1.7% and 6% [4,5]. Our study aimed to assess the prevalence of PE and its effect on outcomes, such as ILD progression, PH, and mortality, in ILD patients at a single center. We also described the PE events using clinical and radiographic features.

## Materials and methods

This retrospective chart review was conducted at King Abdulaziz University Hospital in Jeddah. The author obtained approval from the research ethics committee at the King Abdulaziz University Faculty of Medicine’s Unit of Biomedical Ethics (Reference No. 430-21). Inclusion criteria included patients aged 18 years or older with a diagnosis of ILD who had at least one CTPA scan done. All patients diagnosed with ILD between January 1, 2010, and June 30, 2021, were screened according to the inclusion criteria.

Data collection

Enrolled patients’ medical charts were reviewed to collect the following data: gender, age at ILD diagnosis based on the first chest computed tomography (CT) showing ILD, presence of PE, age at PE diagnosis, time (in months) between ILD and PE diagnoses, and type of the ILD in which the diagnosis was made by the treating pulmonologist, who often discussed clinical, radiographic, and histopathological data in multidisciplinary meetings. We also recorded the following: the presence of ILD radiographic progression as per the reporting radiologist’s opinion on the increase of lung abnormalities if at least six months elapsed between two consecutive chest CT images; the presence of pulmonary hypertension as assessed by echocardiography; type of PE based on radiographic appearance (saddle, segmental, chronic, recurrent) and clinical features (massive); treatment received for PE, presence of risk factors for venous thromboembolism (VTE), and mortality from any cause.

Statistical analysis

The statistical analysis was conducted using Minitab Statistical Software, version 21.1.0 (Minitab Pty Ltd, Sydney, Australia). The continuous variables were measured using means and were compared using two-tailed Student’s t-tests. Categorical variables were reported as counts and percentages and compared using Fisher’s exact tests. A p-value < 0.05 was considered significant.

## Results

Demographic data

A total of 153 patients were diagnosed with ILD between January 1, 2010, and June 30, 2021. Among that group, 48 patients (31.4%) underwent a CTPA and were included in the analysis. Of the 48, seven had PE confirmed by CTPA (14.6%). The mean age at the time of ILD diagnosis based on the first chest CT documenting ILD was 70 ± 9.78 years in the PE group and 61.4 ± 14.42 years in the non-PE group. No statistical difference existed between the two groups regarding age (p = 0.137). The mean age at the time of PE diagnosis was 70 ± 9.73 years. No statistical difference existed between the PE and non-PE groups regarding gender (p = 1) (Table 1). 

**Table 1:** Demographic data ILD: Interstitial lung disease; PE: Pulmonary embolism; N/A: Not applicable Age presented as Mean ± SD

	PE group (7, 14.6%)	Non-PE group (41, 85.4%)	p-value
Age at ILD diagnosis (years)	70 ± 9.78	61.4 ± 14.42	0.137
Age at PE diagnosis (years)	70 ± 9.73	N/A	N/A
Gender (n, %)			1
Male	4, 57.1%	20, 48.8%	
Female	3, 42.9%	21, 51.2%	

PE characteristics

All identified PE events (7) were segmental as demonstrated on the images (100%). No massive PE events (associated with hemodynamic instability) occurred, and one event was saddle PE (14.3%). Recurrent PE was reported in one case (14.3%), and two patients (28.6%) had chronic PE as demonstrated by CTPA. In the PE group, no reported risk factors for VTE were found. Most of the patients (6) received warfarin as an anticoagulant (85.7%), and one received low-molecular-weight heparin (LMWH) (14.3%). No PE events were diagnosed prior to ILD diagnosis, three patients (42.9%) had a simultaneous diagnosis of PE and ILD, and four patients (57.1%) had a PE event diagnosed after ILD by a mean time of eight months (Table 2).

**Table 2:** Characteristics of PE events Total number of patients in the PE group = 7 LMWH: Low-molecular-weight heparin; PE: Pulmonary embolism; ILD: Interstitial lung disease

Characteristics	n, %
Massive PE	0
Saddle PE	1, 14.3%
Segmental PE	7, 100%
Recurrent PE	1, 14.3%
Chronic PE	2, 28.6%
Presence of risk factor for PE	0
Anticoagulant received	
Warfarin	6, 85.7%
LMWH	1, 14.3%
Chronology of PE diagnosis with ILD diagnosis:	
Before ILD	0
Simultaneous	3, 42.9%
After ILD	4, 57.1%

Outcomes

**Figure 1:**
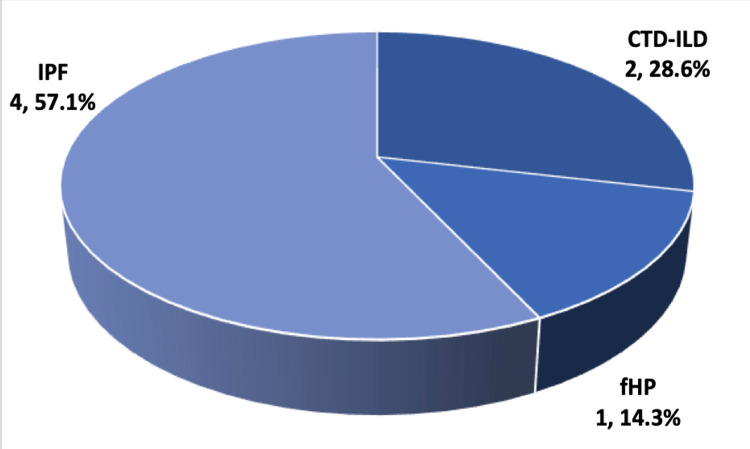
Type of ILD in PE group Data are shown as n, % IPF: Idiopathic pulmonary fibrosis; CTD-ILD: Connective tissue disease-associated ILD; fHP: Fibrotic hypersensitivity pneumonitis

**Figure 2:**
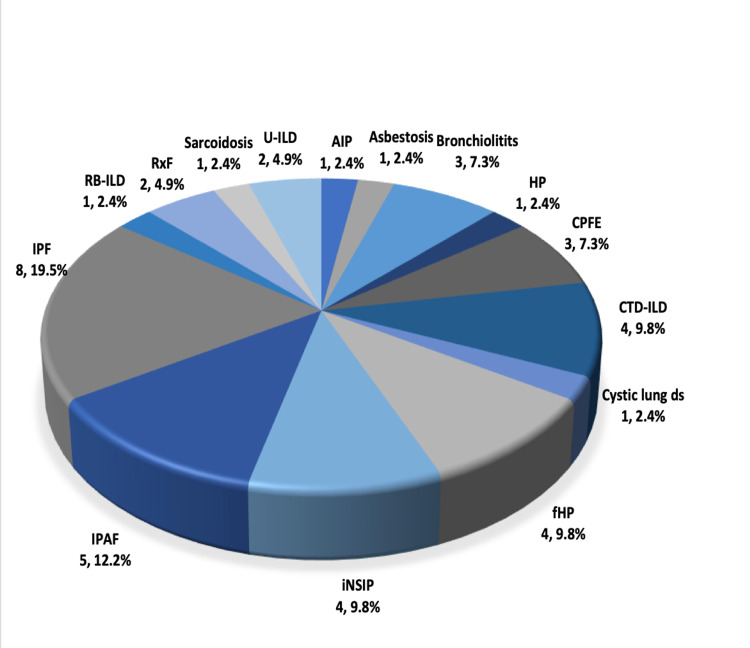
Type of ILD in non-PE group Data are shown as n, %. IPF: Idiopathic pulmonary fibrosis; CTD-ILD: Connective tissue disease-associated ILD; fHP: Fibrotic hypersensitivity pneumonitis; IPAF: Interstitial pneumonia with autoimmune features; RB-ILD: Respiratory bronchiolitis ILD: RxF: Radiation-induced fibrosis; U-ILD: Unclassifiable ILD; AIP: Acute interstitial pneumonia; HP: Hypersensitivity pneumonitis, non-fibrotic); CPFE: Combined pulmonary fibrosis with emphysema; iNSIP: Idiopathic nonspecific interstitial pneumonia

The measurements regarding the outcomes for radiographic progression of ILD, PH, and mortality are presented in Table 3. The available follow-up images to assess ILD progression were available for five patients in the PE group and 29 in the non-PE group; two patients (40%) and 14 patients (48.3%), respectively, showed radiographic progression, and no significant difference existed between the two groups (p = 1). The number of patients who had an echocardiogram done to assess for PH was seven in the PE group and 38 in the non-PE group. We found no difference in the development of PH, as assessed by echocardiography, between the two groups (p = 1). Also, no difference in mortality was found (p = 0.683). The types of ILD diagnoses for both groups are shown in Figures 1, 2. There was no difference between the two groups regarding ILD diagnosis type. 

**Table 3:** Outcome measures ^a^ The number of patients who had more than one chest CT to assess interstitial lung disease (ILD) progression was five in the pulmonary embolism (PE) group and 29 in the non-PE group. The definition of ILD radiographic progression was based on the reporting radiologist’s opinion on the increase of lung abnormalities if a least six months elapsed between two consecutive CT images. ^b^ PH = pulmonary hypertension. The number of patients who had an echocardiogram done to assess for PH was seven in the PE group and 38 in the non-PE group.

Outcome	PE group (7, 14.6%)	Non-PE group (41, 85.4%)	p-value
ILD progression ^a^ (n, %)	2, 40%	14, 48.3%	1
PH^ b^ (n, %)	4, 57.1%	21, 55.3%	1
Mortality from any cause (n, %)	4, 57.1%	17, 41.5%	0.683

## Discussion

Our study showed a prevalence of 14.6% of PE events in ILD cases in our center. More than half of the ILD cases in the PE group were diagnosed with IPF, and the next frequent diagnoses were CTD-ILD and fibrotic hypersensitivity pneumonitis. The PE prevalence in our study is higher than previous estimations, possibly because of the difference in the sample size of the patients who underwent CTPA. An accurate estimation of the prevalence of PE in ILD would require a large cohort study. Generally, the risk of VTE is higher in ILD and, in particular, IPF patients, than in the general population, as shown by a meta-analysis that found a twofold increase of VTE in an IPF population [6]. In addition, previous reports have demonstrated a high risk of VTE, in particular PE, in non-IPF ILD [4]. Luo et al. conducted a prospective study looking at the prevalence of VTE in CTD-ILD and idiopathic interstitial pneumonia and found that approximately a quarter of the enrolled patients developed VTE during the follow-up period and that dyspnea, lower extremity edema, palpitation, and positive D-dimer were associated with VTE risk [7]. Because our study was retrospective, we could not examine the role of applying the validated VTE clinical probability scores on our patients.

Patients with ILD have low respiratory and cardiac reserves, and a PE event is considered a significant burden on their health. Although anecdotal evidence suggests that VTE is associated with poor survival in patients with IPF [8], we did not find a statistically significant difference in mortality between the PE and non-PE groups, perhaps because of the small sample size. Regarding the timing of PE in relation to ILD diagnosis in our study, no PE events were diagnosed before the development of ILD. Less than half of the patients were diagnosed with ILD and PE simultaneously upon presenting with an acute PE event, and just over half were diagnosed with PE after ILD, with a mean time of eight months. This observation is not representative of an accurate timeline between ILD diagnosis and a PE event because the patients in our study did not have a CTPA when first presenting with ILD, and PE events could have been missed. Studying the time between VTE and ILD diagnosis in a large cohort would be helpful for understanding the potential pathogenesis of VTE in this subset of patients.

In our study, all PE cases had no known risk factors for VTE, indicating that the presence of ILD, regardless of the underlying etiology, might be a risk factor for PE. These patients might have limited daily activity and deconditioning secondary to their respiratory compromise, possibly adding to the risk of PE events [9]. Another explanation of the pathogenesis of VTE in ILD could be the presence of common pathogenetic pathways between ILD and VTE supported by the enhanced prothrombotic state seen in IPF patients [10,11]. In addition, Sode et al. found a high risk of subsequent ILD diagnosis in patients ever diagnosed with PE, and these results again raise the point of whether a common pathogenetic pathway between VTE and ILD or a causality relation exists in which PE leads to a local trigger of inflammation and fibrosis cascade in the lung parenchyma [12]. In our study, we found two cases of CTD-ILD that developed PE. CTD-ILD is also associated with an increased risk of VTE, given the underlying inflammation and autoimmunity mechanism, further contributing to the pathogenesis of VTE in ILD cases other than IPF [7].

We were not able to find a significant statistical difference between the PE and non-PE groups regarding the progression of ILD, development of PH, or mortality. PH is not an uncommon complication of ILD and can be related to underlying lung disease (group 3 PH), pulmonary arterial hypertension (group 1 PH) in cases of CTD-ILD, concomitant cardiac dysfunction (group 2 PH), and chronic thromboembolic PH (CTEPH; group 4 PH), the latter has scarcity in the literature regarding its prevalence in ILD patients. Most of the patients were treated with warfarin as an anticoagulant, and given the concerns about the negative effect of warfarin on survival in patients with IPF [13], we should be cautious in extrapolating that data to IPF patients who require warfarin for underlying reasons, such as VTE, as this group of patients was excluded from that study.

Study limitation

This study was retrospective in a single center with a small number of patients who underwent CTPA, thus the results might not be representative of the actual prevalence of PE in ILD. We did not evaluate confounders, including comorbidities. Evaluating the clinical outcomes in event of PE in ILD, such as the development of PH, CTEPH, AEILD, and mortality, requires a large longitudinal study to accurately estimate the outcomes.

## Conclusions

Our result showed a high prevalence of PE in ILD patients which is a major comorbidity that can have a significant impact on morbidity and mortality. We did not reach a conclusion regarding factors associated with increased PE risk, yet we were able to characterize the PE events and examined its time relation with ILD diagnosis helping us to understand the clinical presentation of this condition in ILD cohort. Given the complexity of ILD cases and the varying degrees of respiratory compromise these patients have, it is of utmost importance to have a low threshold for evaluating PE in the event of a change in respiratory status, in particular before labeling the patients with AEILD, which has a worse clinical course and outcome than PE.
